# *Plasmodium falciparum* Erythrocyte Invasion: Combining Function with Immune Evasion

**DOI:** 10.1371/journal.ppat.1003943

**Published:** 2014-03-20

**Authors:** Gavin J. Wright, Julian C. Rayner

**Affiliations:** 1 Cell Surface Signalling Laboratory, Wellcome Trust Sanger Institute, Wellcome Trust Genome Campus, Hinxton, Cambridge, United Kingdom; 2 Malaria Programme, Wellcome Trust Sanger Institute, Wellcome Trust Genome Campus, Hinxton, Cambridge, United Kingdom

## The *Plasmodium falciparum* Merozoite: A Dedicated Invasion Machine

**Figure 1:**
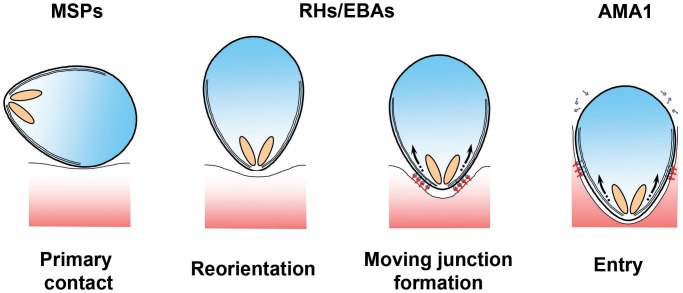
Erythrocyte invasion is a complex multistep process. The different stages of erythrocyte invasion are drawn in cartoon form. The different protein families discussed in this review are thought to operate at different steps during invasion, with MSPs functioning at the very earliest stages, PfRH and PfEBAs functioning during the formation of a tight contact between the merozoite apex and the erythrocyte surface, and the AMA1–RON interaction being tightly associated with the moving junction itself [4], [17]. Detailed reviews of the molecular and ultrastructural basis of invasion are available in other reviews [15]–[17], [24].

Throughout their extraordinarily complex life cycle, *Plasmodium* parasites must navigate a wide range of intracellular and extracellular environments in both vertebrates and invertebrates. To achieve this, the parasite develops into a series of distinct morphological forms or “zoites,” each of which is specialised for a particular biological challenge. Merozoites—ovoid cells approximately 1 µm long that are released from an infected erythrocyte once development is complete—are the epitome of a specialised *Plasmodium* stage. Merozoites do not replicate outside of their host: they exist purely to find and invade erythrocytes. To do so, they undergo a series of complex manoeuvers, first visualised by pioneering video microscopy and electron microscopy studies more than 30 years ago [1], [2]. Initial contacts between the merozoite and erythrocyte can occur at any point on the merozoite surface, which are rapidly followed by the reorientation of the polar merozoite such that its apical end directly apposes the erythrocyte membrane (see Figure 1). This allows the parasite to deploy a series of specialised apically located secretory organelles: rhoptries, micronemes, and dense granules. These organelles then discharge their contents in a regulated and ordered schedule during and immediately after the invasion process at the site of contact [3]–[5]. Ligands released in this manner interact with erythrocyte surface receptors to form an electron-dense thickening of the erythrocyte membrane at the nexus of erythrocyte–merozoite contact. The junction is passed around the merozoite surface in a belt-like structure, driven by an actin-myosin motor that is anchored to the merozoite's inner membrane complex (IMC), which contributes to the formation and maintenance of the merozoite's characteristic ovoid shape [6], [7]. Invasion is completed as the moving junction closes behind the merozoite in the fashion of an iris diaphragm, leaving the merozoite enclosed within a parasitophorous vacuole.

**Figure 2:**
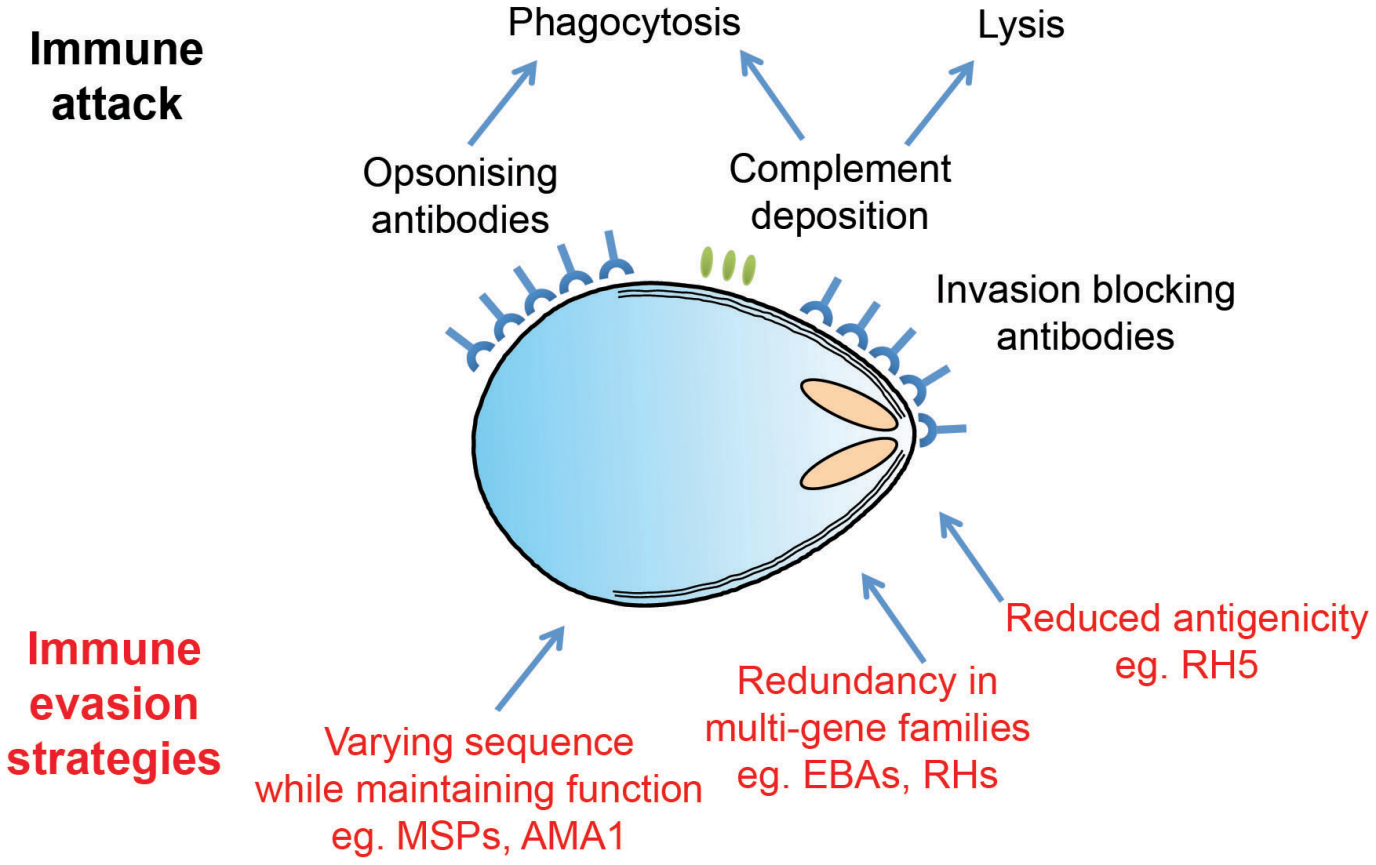
*Plasmodium* merozoites face an array of immunological challenges. Merozoites are the only extracellular stage of the *Plasmodium* life cycle and are therefore exposed to an array of immune attack mechanisms, as illustrated in cartoon form. Merozoite antigens are known to be the target of antibody responses, which operate both by opsonisation leading to phagocytosis and by simple steric hindrance of receptor–ligand interactions critical for invasion. Complement deposition on the merozoite surface may also play a role in parasite clearance. To avoid these attack mechanisms, *Plasmodium* parasites have evolved a number of distinct evasion responses. Some merozoite antigens such as AMA1 are highly polymorphic, while members of the PfRH and EBA multigene families are largely redundant and have variable expression profiles. Both of these strategies slow the development of protective immunity by forcing the antibody response to efficiently recognize multiple targets in order to mount an effective response. Finally, RH5 appears to be poorly immunogenic in the context of natural infections, perhaps due to limited levels of expression and exposure.

The overall process of invasion may be complex, but it is also extremely rapid. A series of recent studies all concur that invasion is complete, on average, less than two minutes after merozoites are released [8], [9]. Why the need for speed? The answer likely lies in the fact that the merozoite is one of the few stages of the *Plasmodium* life cycle in which the parasite is extracellular and therefore directly exposed to immunological attack (see Figure 2). To survive, the parasite must restrict its window of exposure to minimise neutralization by complement mediated lysis or opsonisation by host-derived antibodies. Speed alone, however, is not enough, and the merozoite also deploys an array of escape mechanisms to keep the immune system at bay long enough to complete the invasion process. Understanding these mechanisms is more than simply an interesting biological question. Because erythrocyte invasion is an obligate part of the parasite's lifecycle, blocking invasion should prevent parasite growth, making invasion an attractive vaccine target. However, vaccine trials targeting invasion have faltered, most likely because they have been countered by one or more of the merozoite's immune-evasion mechanisms. It is only by understanding the parasite's immunoprotective mechanisms that we can hope to identify and exploit weak points that could be targeted by a vaccine.

## Distraction through Diversity

Given the complexity of the invasion process, it is no surprise that the merozoite expresses a diverse array of invasion-associated proteins. The combination of genome sequencing [10], large-scale gene [11], [12] and protein profiling studies [13], [14], together with the rapid expansion in *P. falciparum*–experimental genetic technologies [15] have identified more than 50 *P. falciparum* proteins that are hypothesised to somehow be involved in the invasion process, although in the vast majority of cases their precise function is unknown. The most well-studied of these have been organised into distinct functional classes: MSPs (merozoite surface proteins), which form a structurally complex coat around the merozoite surface, and the PfEBAs (*P. falciparum* erythrocyte binding antigens, related to the *P. vivax* duffy binding protein) and PfRHs (*P. falciparum* reticulocyte binding protein [RBP] homologues, related to the *P. vivax* RBPs [PvRBPs]), which are stored in specialised apical organelles, the rhoptries and micronemes [16], [17].

PfRHs and PfEBAs are generally thought to function later during invasion, and at least some members may be released on to the merozoite surface in a regulated manner after the initial merozoite–erythrocyte contact has been made [5], [18]. MSPs, by contrast, are thought to function during the initial contact phases of invasion and are exposed to antibodies as soon as the merozoite is released into the bloodstream. To avoid the host immune response, many MSPs are highly polymorphic, and MSP genes frequently bear signatures of being under balancing selection pressure [19], [20], resulting in the simultaneous circulation of multiple variants of the same gene within a population. Several of the most abundant MSPs, such as MSP1 and MSP2, are diallelic, with multiple variants found within each allelic class. Other MSPs are part of multigene families, and in some cases, such as the 6-cys proteins, a clonally variant expression system results in the expression of different members of each family in different parasite lines [20]. Together, these diversity-generating mechanisms can result in immunologically distinct merozoites within a single infected individual, especially if they are simultaneously infected with multiple, genetically distinct strains. In such a circumstance, even a primed immune system is unlikely to effectively block the invasion of all merozoites within their fleeting period of extracellular exposure.

This distraction-through-diversity approach is highly effective. Host antibody responses to MSPs are often very strong in adults who have been previously infected with *P. falciparum* on multiple occasions, and the anti-merozoite immune responses that they generate are known to be able to reduce the effectiveness of parasite invasion [21]. Despite this, immunity to *P. falciparum* is only ever partially effective, with populations of parasites continuing to multiply even within adults who are clinically immune—their immune responses may limit symptoms, but are not sufficient to eradicate parasites. This stark fact highlights the challenge facing the development of invasion-blocking vaccines. It could theoretically be possible to develop multivalent vaccines that target multiple genetic variants of a given MSP, but the outcome, at best, is likely to only ever recapitulate natural immunity—a partial block that might be sufficient to prevent disease (itself, a worthy goal) but is unlikely to be sufficient to prevent infection or contribute significantly to the goal of malaria eradication. It should be noted that although high levels of diversity are the rule for MSPs, subdomains of specific MSPs can be highly conserved and, therefore, have potential as vaccine targets. The C-terminal domain of MSP1, MSP1-19, is by far the most well-studied example of this, and antibodies that target MSP1-19 can have potent invasive inhibitory effects [22]. Despite these attractive features, Phase IIb trials of a region of MSP1 that includes MSP1-19 were disappointing, suggesting that a more in-depth understanding of this target is necessary [23].

## Elucidating the Molecular Mechanisms of Invasion Reveals Redundancy

Not only are MSPs often highly polymorphic, making them challenging targets, they also generally have poorly defined functions. A more rational approach would be to use a mechanistic understanding of the parasite and host molecules involved in invasion to identify targets for a potential invasion-blocking therapeutic. For many years, invasion research has focused on the role of the PfRHs and PfEBAs, and this work has led to significant advances in mechanistic understanding [16], [24]. However, the potential of PfRHs and PfEBAs as intervention targets has been compounded by another evasion mechanism used by the parasite—functional redundancy. It has been known for some time that *P. falciparum* merozoites can use several alternative pathways to invade human erythrocytes. The definition of what exactly constitutes an “alternative invasion pathway” is not clear, and the area in general is in urgent need of a systematic overhaul and agreement on terminology. A simple and pragmatic definition is that when the repertoire of available erythrocyte receptors is restricted in vitro either by enzyme treatment (generally with trypsin, chymotrypsin, or neuraminidase) or through the use of erythrocytes from human donors with defined blood groups, there can be a range of phenotypic outcomes depending on the *P. falciparum* strain. Culture-adapted *P. falciparum* strains have long been known to have differential abilities to invade both enzyme-treated erythrocytes [25] and erythrocytes from individuals that lack expression of specific surface receptors [26], [27]. Similar observations have been made using field isolates, both in strains that have recently been adapted to in vitro culture [28], and in parasites that have never been adapted but were phenotyped in their first round of invasion in vitro [29], [30]. A large body of experimental data suggests that it is the PfRH and PfEBA protein families that are responsible for this functional redundancy: when individual ligands in these families are genetically deleted, a change in the ability of the parasites to invade enzyme-treated erythrocytes is the most commonly observed phenotype [31]–[34]. Similar effects can be observed by the addition of antibodies directed to the RHs (reticulocyte binding protein homologues) or EBAs (erythrocyte binding antigens) in parasite growth assays [35]–[37].

**Figure 3:**
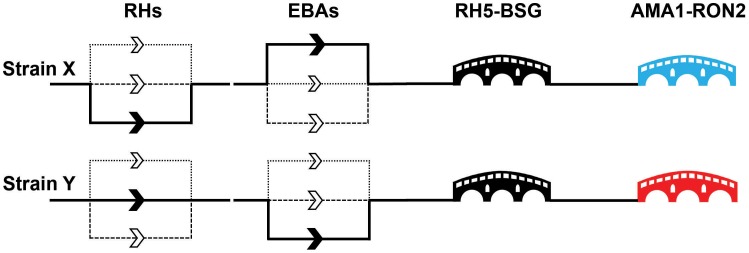
A molecular understanding of invasion leads to the identification of critical target points. The invasion events controlled by the paralogues within the EBA and RH families are thought to be redundant with the relative importance of individual genes differing between strains. This leads to a model of invasion where there are a number of parallel “alternative invasion pathways,” as indicated by multiple routes in the diagram for two nominal strains (X and Y). The differential dependencies on particular EBA and RH paralogues is indicated by the weighting of the line, with the unbroken line representing a major dependency and the dashed and dotted lines nonpreferred pathways for that strain. By contrast, the nonredundant RH5–basigin and AMA1–RON2 interactions are represented by critical “bridges.” The immunogenic AMA1 protein is highly variable between strains and is therefore represented by different colours: neutralising host antibodies elicited by one AMA1 variant would not protect against a strain containing a different AMA1 variant. In natural infections, RH5 is not immunogenic, suggesting that the parasite has protected this critical stage by an immunomodulatory mechanism.

It is almost certain that the parasite has evolved this functional redundancy in invasion ligands to counter the host humoral immune response. As noted above, members of the EBA and RH family are known to be targets of host antibodies, and so if the parasite relied on a single ligand for the later stages of invasion, the host could logically acquire sterile protective immunity to the parasite. The parasite's answer to this seems to have been to expand these two protein families, creating multiple paralogues and thereby presenting the host immune system with the more complex problem of blocking several ligands. The relative functional weighting on any one particular EBA or RH ligand is presumably governed by many factors, including their relative expression levels [29], [30], [33], differences in amino acid sequences between strains [38], [39], and the host genotype and immunological status (Figure 3). Parasite genetic background certainly has a major impact on the importance of a given receptor–ligand interaction. This is most starkly apparent in the case of PfEBA and PfRH genetic knockouts, where PfEBA175 deletion results in a major shift away from neuraminidase sensitive invasion in W2mef but not 3D7 [40], [41]. PfRH1 deletion also has differential effects depending on the strain [41]. This implies a hierarchy of interactions within the PfRH and PfEBA paralogues, with the relative importance of each interaction being strain-specific, as has been elegantly proposed by previous papers [32], [34], [42].

While the overall point that variation in PfRH and PfEBA ligands is the explanation for redundancy in invasion pathways, we believe that a few notes of caution are needed, especially when interpreting in vitro genetic studies. PfRH and PfEBA ligands are often discussed as if they are completely functionally interchangeable, but this is likely to be an oversimplification. Both gene families are deeply phylogenetically rooted and all *Plasmodium* genomes sequenced to date contain at least one member of each family. If they were precisely functionally equivalent, it would be expected that one family would have been lost in at least one *Plasmodium* species, but instead most genomes actually have an expansion of one or both families, with multiple paralogues present. Their functions may therefore be subtly distinct; for example, it is proposed that the primary function of the PfRHs is to propagate a signal that triggers the subsequent release of the PfEBAs [24]. However, while PfRH and PfEBA ligands may not be strict functional alternatives, some members are certainly able to compensate for each other in particular contexts, at least in vitro, such as the up-regulation of *RH4* transcription in *EBA175* knockout lines [33].

The expansion of the EBA and RH families in *P. falciparum* and the consequent redundancy and complexity that it creates means that several of these ligands would have to be simultaneously targeted to effectively reduce or block all invasion. This makes the development of such a vaccine technically challenging, although initial trials with PfRH and PfEBA combinations do show some promise [32], [43], [44]. However, while such combinations may be viable vaccines, the extent of natural diversity in the expression and sequences of PfRH and PfEBA means that they will need to be tested against a very wide range of natural isolates before their potential can be truly assessed, and these trials have not yet been conducted. It also remains to be seen whether clinical trial funders will be prepared to meet the higher manufacturing cost of a multivalent vaccine. Furthermore, it is worth pointing out that the design of some Phase IIa vaccine trials where subjects are experimentally infected can set a very high bar for efficacy [45]. In these trials, curative drug treatment must be applied as soon as parasites are detected by PCR in the blood, for quite understandable health and ethical reasons. This means that the trial tests parasite multiplication rate, rather than protection from symptoms that would be the more likely outcome of multivalent EBA/RH vaccines. Vaccines could therefore conceivably fail in such a Phase IIa trial, but still provide significant symptomatic protection in a natural infection. It is therefore likely that we will either have to abandon these complex targets, or change the design of vaccine trials to allow us to better detect their effects, for example, by including dose escalation studies to enable the detection of effects that may be visible only at low starting parasitemias.

## Identifying the Critical Bridges during Invasion

Are there any other invasion ligands that could be targeted that avoid the problem of PfRH and EBA redundancy? So far, there are two parasite ligands that can be targeted by antibodies to induce a potent block in invasion and also appear to be essential and nonredundant, as attempts to genetically delete them have failed. Both have known receptors: PfRH5 and its erythrocyte receptor, basigin, and AMA1 (apical membrane antigen) and its parasite-encoded receptor, RON2 (rhoptry neck protein) (see Figure 3).

### AMA1–RON2

AMA1 is undoubtedly an important parasite invasion ligand. Readily identifiable AMA1 orthologues exist across the genus *Apicomplexa*, and genetic deletion experiments have largely shown that they are essential [46], [47], although the recent report that AMA1 is not absolutely required for invasion by *P. berghei* merozoites will require detailed follow-up using tightly regulatable systems in other *Plasmodium* species [48]. AMA1 is a micronemal type I transmembrane protein that translocates to the surface of invasive zoites, including the *P. falciparum* merozoite [49], and is localised at the moving junction during invasion. Its precise function during erythrocyte invasion is not entirely clear and has been proposed to play a role in merozoite reorientation [50], erythrocyte binding [51], [52], invasion efficiency [53], rhoptry secretion [54], [55], and formation of the moving junction [4], [56], [57]. Copurification experiments [57], [58] and subsequent structural studies [59], [60] have led to a model in which the parasite inserts its own AMA1 receptor complex into the target cell membrane. The RON complex (RON2, 4, 5, and 8) are secreted from the rhoptries, and the RON2 protein presents a surface-exposed loop that is inserted into a hydrophobic groove in the AMA1 ectodomain, thereby providing a receptor–ligand pair for invasion. This model is supported by functional data that demonstrate that either antibodies against AMA1 [61], [62] or short, soluble peptides that bind in or near the groove block invasion [60], [63].

Given its important role in parasite invasion, AMA1 has been a high priority blood-stage vaccine candidate for many years [62], [64], but the general conclusion from multiple trials has been that vaccine-induced invasion-blocking antibody responses to AMA1 are strain-specific and therefore provide protection only to vaccine-homologous parasite strains [65], [66]; that is to say, only strains that encode an AMA1 variant immunologically similar or identical to the AMA1 protein sequence variant used in the vaccine are inhibited. Interestingly, this means that the AMA1 protein has the remarkable property of retaining its functional role during invasion whilst tolerating many sequence variants that are immunologically distinct. It therefore appears that the parasite protects AMA1 by evolving a spectrum of variants to create an ever-moving target which is difficult to vaccinate against, similar to the MSPs. This makes the AMA1–RON complex a challenging vaccine target, albeit one that is potentially solvable by the inclusion of multiple AMA1 variants [67] or by targeting the RON complex, rather than AMA1 itself.

### RH5–basigin

Recently, another merozoite–erythrocyte interaction was identified that also appears to function as an essential pinch point in the invasion pathway: that between the parasite ligand RH5 and the Ok blood group antigen, basigin. Originally identified by analysing the *P. falciparum* genome sequence [68], RH5 was grouped into the RH family of parasite ligands by the presence of some—albeit limited—sequence homology [69]. RH5 differs from other PfRH family members because it is much smaller and is predicted to be secreted rather than anchored to the parasite membrane, and it is known to interact with another secreted parasite protein, RIPR [70]. Like AMA1, RH5 is also localised to the moving junction during invasion [71] and attempts to genetically delete *RH5* in several strains were unsuccessful [68], [71], suggesting it played an important role in blood stage growth. Its essentiality in the invasion process was reinforced by the identification of its receptor, basigin [72], using a protein interaction screening method called AVEXIS (avidity-based extracellular interaction screen) [73]. Importantly, monoclonal antibodies against the basigin receptor were able to completely block all detectable invasion across a panel of different laboratory-adapted parasite strains and recent field isolates [72]. Unlike AMA1, identifiable orthologues of PfRH5 have only been identified in one closely related species, *P. reichenowi* [74], and not in the other major human malaria species such as *P. vivax*. *RH5* also differs from *AMA1* in that its polymorphism is very limited within *P. falciparum* populations, suggesting that it is not under significant immune selection pressure [75], [76]. Consistent with this, polyclonal or monoclonal antibodies raised against RH5, either using a viral delivery system [77] or using a recombinant RH5 protein expressed using either eukaryotic or prokaryotic expression systems [76], [78], [79], are able to prevent parasite growth in vitro. Importantly, and by contrast with AMA1, this blocking effect is effective across multiple strains of parasite, including those parasite strains that contain the most frequently observed polymorphisms in *RH5* globally [76].

This raises an apparent paradox. Naturally acquired immunity to malaria is typically not sterile, yet antibodies against RH5 can potently block invasion across multiple strains; that is, they should be able to provide sterile protection. One would, therefore, logically infer that clinically immune adults should not have high titres of anti-RH5 antibodies, because if they did, they would be sterilely protected and lack detectable parasitemia. Although studies of anti-RH5 responses are limited, anti-RH5 responses were low in Kenyan adults and showed no evidence of age-dependent acquisition [77]; only 15% of serum samples from Senegal were seropositive for anti-RH5 responses [80], while in a large comparative study in Papua New Guinea, RH5 had one of the lowest seropositivity rates of the 91 merozoite proteins tested [81]. Despite their relatively low prevalence, anti-RH5 antibodies purified from human immune serum had strong invasion inhibitory effects [80], and in a time-to-reinfection study in Mali, the presence of anti-RH5 antibodies was strongly associated with protection from malaria episodes [82]. While much more work is clearly needed, the lower levels of anti-RH5 response observed to date suggests that the parasite has evolved a third mechanism to evade host immune responses to a critical point in the invasion pathway, distinct from the redundancy and polymorphism that protect other invasion proteins: the ability to produce a protein that is not—*at least in the context of a natural infection*—immunogenic. The mechanism by which the RH5 protein is able to evade the human humoral immune response is currently not known, but it could be due to limited levels of expression and exposure or through an active immunomodulatory mechanism involving a direct interaction with additional host proteins. Importantly for the use of RH5 as a vaccine, this ability to evade the host antibody responses appears to be context-specific since high-titre antisera can be raised to a recombinant RH5 protein/adjuvant mix in rabbits [76] and mice [77]. However, the apparently low immunogenicity of native RH5 may prevent significant levels of natural boosting following vaccination, which could affect the induction or longevity of any RH5 vaccine-induced response. Nonetheless, the pan-strain dependency on the interaction of RH5 with basigin for invasion and its susceptibility to elicited antibodies make RH5 a highly promising target for a blood-stage vaccine, either alone or in combination with other synergistic targets [83], and further trials are clearly justified.

## Conclusions and Future Directions

Given that it is essential for the survival of blood-stage parasites, erythrocyte invasion has long been viewed as a point in the life cycle that could be rationally targeted in the development of an anti-malarial vaccine. Although vaccine development priorities have recently become focused primarily on transmission blocking and pre-erythrocytic stages, the development of the RTS,S vaccine reinforces the fact that vaccines directed at a single target are never likely to be 100% effective. Furthermore, a highly effective blood-stage vaccine will, by definition, affect transmission by reducing the pool of ring-stage parasites capable of gametocyte differentiation. It is our strong opinion that invasion targets must be considered as crucial components of any second-generation multistage malaria vaccine. However, one of the very features of invasion that make it an attractive vaccine target—its exposure to the antibody-mediated immune response—also makes it a difficult target, because the host–parasite “arms race” has forced the parasite to evolve sophisticated immunoprotective mechanisms to shield itself. In particular, the parasite has protected the MSPs and RH/EBA ligands by generating sequence diversity and functional redundancy, resulting in parasites that use experimentally definable alternative invasion pathways that are difficult to target. One possible way to circumvent this problem would be to generate a multicomponent vaccine that attempts to neutralise all alternative ligands, but this is likely to be expensive to manufacture, and at best may simply recapitulate the partial protection found in clinically immune adults. Nonredundant interactions essential for invasion (AMA1–RON2 and RH5–basigin) make conceptually more attractive targets, but perhaps unsurprisingly, the parasite has evolved mechanisms to protect these critical invasion ligands. Intriguingly, however, the parasite protects AMA1 and RH5 from the host immune response by different mechanisms: it protects AMA1 by creating a series of immunologically distinct variants, while native RH5 appears immunoprotected. Critically, RH5 does not appear to be intrinsically nonimmunogenic since high antibody titres to RH5 are readily obtained in experimental models, and can potently inhibit invasion in vitro [76], [77]. This raises the possibility that the parasite's immunoprotective mechanisms could be circumvented by eliciting unnatural immunity with an RH5-based vaccine. One of the primary challenges in RH5 vaccine development will clearly be identifying adjuvants that raise sufficiently high antibody titres in the absence of immune boosting. However given the lack of success to date in circumventing the merozoite's immune evasion mechanisms, developing a potent anti-RH5 immune response and identifying other targets that functionally synergise with such responses represents the current best hope for an invasion-blocking vaccine.
